# Current Operating Procedure (COP) for Bleomycin ElectroScleroTherapy (BEST) of low-flow vascular malformations

**DOI:** 10.2478/raon-2024-0061

**Published:** 2024-11-28

**Authors:** Tobian Muir, Walter A Wohlgemuth, Maja Cemazar, Giulia Bertino, Ales Groselj, Lakshmi A Ratnam, Ian McCafferty, Moritz Wildgruber, Bernhard Gebauer, Francesca de Terlizzi, Alessandro Zanasi, Gregor Sersa

**Affiliations:** South Tees NHS Foundation Trust, Middlesbrough TS4 3BW, United Kingdom; Clinic and Policlinic of Radiology, Martin-Luther University Halle-Wittenberg, Halle (Saale), Germany; Department of Experimental Oncology, Institute of Oncology Ljubljana, Ljubljana, Slovenia; Faculty of Health Sciences, University of Primorska, Isola, Slovenia; Department of Otolaryngology Head Neck Surgery, University of Pavia, Istituto di Ricovero e Cura a Carattere Scientifico (IRCCS) Policlinico San Matteo Foundation, Pavia, Italy; Department of Otorhinolaryngology and Cervicofacial Surgery, University Medical Centre Ljubljana, Ljubljana, Slovenia; Faculty of Medicine, University of Ljubljana, Ljubljana, Slovenia; St George’s University Hospitals NHS Foundation Trust, London, United Kingdom; City St George’s University of London, School of Health & Medical Sciences, London, United Kingdom; Birmingham Women’s and Children’s Hospital NHS Foundation Trust, Birmingham, United Kingdom; Department of Radiology, University Hospital, LMU Munich, München, Germany; Interdisziplinäres Zentrum für Gefäßanomalien (IZGA), University Hospital, LMU Munich, München, Germany; Diagnostic and Interventional Radiology, Charité Universitätsmedizin Berlin, Germany; IGEA S.p.A., Clinical Biophysics Lab. Carpi, Modena, Italy; Faculty of Health Sciences, University of Ljubljana, Ljubljana, Slovenia

**Keywords:** low-flow vascular malformations, bleomycin sclerotherapy, bleomycin electrosclerotherapy, BEST

## Abstract

**Background:**

Bleomycin ElectroScleroTherapy (BEST) is a new approach in the treatment of vascular malformations. After bleomycin is administered to the malformation, electric pulses are applied to the target area to enhance the effectiveness of bleomycin. The mode of action is comparable to the effect of electrochemotherapy on tumour vasculature. For the wider and safer use of BEST in the clinical treatment of low-flow vascular malformations, this Current Operating Procedure (COP) is being prepared. It is a proposal for the clinical standardisation of BEST using the Cliniporator^®^ as the electrical pulse generator with its associated electrodes. The electrical parameters considered in this protocol are those validated by the European Standard Operating Procedures for Electrochemotherapy (ESOPE) with the Cliniporator^®^.

**Conclusions:**

General requirements are proposed, and, depending on the type of lesion, local skills and the availability of radiological equipment, two technical approaches of BEST are described based on ultrasound guided intervention or combined ultrasound and fluoroscopic guided intervention.

## Introduction

Reversible electroporation has several biomedical applications. By enhancing the delivery of drugs or nucleic acids to normal or tumour tissues, it can be used in oncology for tumour treatment, for vaccination or, as recently described, for Bleomycin ElectroScleroTherapy (BEST) of vascular malformations. There are several clinical reports published on the use of BEST in the treatment of low-flow and high-flow vascular malformations.^1,2,3,4,5,6,7,8,9,10^ This approach has gained interest because bleomycin is already widely used in conventional sclerotherapy of vascular malformations and the application of electrical pulses potentiates its effectiveness.^11,12,13,14,15^ Therefore, in BEST treatment, the dose of bleomycin and number of sessions needed could be reduced as compared to conventional bleomycin sclerotherapy, contributing to the safety of the treatment approach. Several case series have been published in adults and children in the last few years, showing that BEST could increase the efficacy of bleomycin and reduce the number of treatments required when treating vascular malformations.^2,4,5,6^ Furthermore, the effectiveness is significant even in patients presenting with therapy-resistant venous malformations.^3^ A recently published paper by Schmidt et al. has demonstrated the safety and effectiveness of BEST in a very large population of 233 children and adults, demonstrating greater efficacy in children.^7^

Why is BEST effective especially on abnormal vasculature? The principle of BEST can be related to the vascular disruption and vascular locking effects of electrochemotherapy. Electrochemotherapy is based on the application of electric pulses to tumours after intravenous or intratumoral bleomycin injection. A substantial portion of the antitumor effectiveness of electrochemotherapy is attributed to its vascular effects.^16^ The application of electric pulses acts on the blood vessels, among other things inducing temporary vasoconstriction. This causes a temporary cessation of blood flow, the so-called **vascular lock effect**. This results in a prolonged retention of injected bleomycin in the region where the electric pulses have been applied, contributing to the effectiveness of electrochemotherapy and BEST. Another feature is the **vascular disrupting effect** due to the increased uptake of bleomycin in endothelial cells to the reversible electroporation. These cells die slowly by apoptosis due to bleomycin-induced G2M-arrest when proliferating. The applied electric pulses temporarily permeabilize the endothelial cell lining, which may further increase bleomycin uptake. The vascular disrupting effect was shown to be predominantly confined to abnormal vasculature in tumours, due to the higher proliferation rate of endothelial cells, compared to normal vasculature.^17,18,19,20,21^ The same could be assumed for the atypical vasculature of low-flow vascular malformations, which are caused by mosaic mutations in the same cellular pathways as in tumour endothelial cells.^22^ The clinical results of BEST are compelling^1^, but its cellular and immunological mechanisms on the dysplastic vasculature need to be explored in further detail to support increased use of BEST in clinical practice and also to provide rationale for its refinement.

The group of clinicians within the InspECT consortium, in collaboration with other experts in the field of vascular malformations treatment, have prepared this document, a Current Operating Procedure (COP). It is a proposal for the clinical standardisation of BEST using the Cliniporator^®^ as the electrical pulse generator with its associated electrodes. The electrical parameters considered in this protocol are those validated by the European Standard Operating Procedures for Electrochemotherapy (ESOPE) with the Cliniporator^®^.^23^ After validation of these COP in the clinical application, a standard operating procedure would need to be prepared.

## Current Operating Procedure (COP)

### Requirements for performing a safe procedure

- Experience or training in the technique of image-guided sclerotherapy.
- Experience or training in the electroporation technique using a Cliniporator^®^ pulse generator.
- Agreed local protocol for the safe administration, use and disposal of a cytotoxic chemotherapeutic agent (bleomycin).
- Agreed local protocol for respiratory monitoring of patients receiving BEST (may be different in children and adults).

### Patient referral and suitability

- Patient referral by a vascular-malformation multidisciplinary team (MDT) or experienced centre.

### Indications for BEST of vascular malformations

- Patients with a low-flow vascular malformation (venous, lymphatic, capillary, mixed type) suitable for BEST, i.e.: injection of bleomycin and safe placement of electrodes into the vascular malformation are technically feasible.
- Patients with a low-flow vascular malformation poorly responding or recurring after previous treatment(s).

### Contraindications for BEST of vascular malformations

- Pregnancy and lactation.
- In adults, previous bleomycin exposure with a cumulative dose greater than 100 000 IU.
- In children, previous bleomycin exposure greater than 1300 IU/kg (taking into account the increasing weight of the child).
- In case of abnormal respiratory results/chest pathology (including previous severe or long COVID) in consultation with a pulmonologist, special care is required, and bleomycin exposure may be contraindicated.
- In patients with impaired renal function, the dose of bleomycin should be reduced at least by 1/3.
- Known allergy or hypersensitivity to bleomycin.
- Presence of significant central venous drainage precluding sclerotherapy.

### Pre-treatment investigations

- **Respiratory surveillance**
  - Follow local procedure.
- **Recommendations for treatment of adults**
  - Agreed protocol for respiratory monitoring.
  - Pre-treatment pulmonary function test and diffusing capacity for carbon monoxide (DLCO) according to local protocol.
  - In the event of abnormal pre-treatment lung function test or DLCO, or known chest pathology (excluding controlled asthma), the patient should be assessed by a pulmonologist.
  - Post-treatment assessment and protocol if patient develops respiratory symptoms need to be established.
- **Recommendations for treatment of children**
  - Further caution is mandatory as in children lungs are still developing. Patients are suggested to be assessed by a paediatric pulmonologist or as per local protocol before BEST treatment. Preassessment for general anaesthesia or sedation.
  - Follow local procedure.
- **Pre-treatment imaging investigation**
  - Ultrasound imaging to determine characteristics, extent and flow status.
  - Magnetic Resonance Imaging (MRI) in most cases prior to interventional treatments to fully define and assess the low flow malformation (extent, deeper parts, drainage, differential diagnosis, multifocal lesion, etc.)
  - Magnetic Resonance Angiography (MRA)/Magnetic Resonance Venography (MRV) may be added.
- If there are concerns about medical history or any abnormal/atypical findings on clinical examination or imaging: Perform an open biopsy or core ultrasound needle-guided biopsy for differential diagnosis.
- For large-volume venous malformations, assess coagulation profile (D-Dimer; fibrinogen) to exclude Localized / Disseminated Intravascular Coagulopathy (LIC/DIC); assess the need for preoperative Low Molecular Weight Heparin treatment (LMWH).

### Patient information

- Discuss the BEST procedure and any possible alternatives.
- Clearly state that bleomycin for vascular malformations is an off-label use as it is in conventional sclerotherapy.
- Provide full information on all possible risks and benefits for the patient to consider (letter/website).
- Discuss the possibility that bleomycin carries a potential risk of pulmonary toxicity.
- Provide information about possible effects of bleomycin on fertility and pregnancy.
- Discuss the potential risk of air embolism if bleomycin is foamed.
- Discuss risks of skin hyperpigmentation.
- Provide contact details in case the patient requires further information.
- Sign informed consent of the patient for the BEST procedure.

### Anaesthesia (follow local procedure)

**FIGURE 1.**
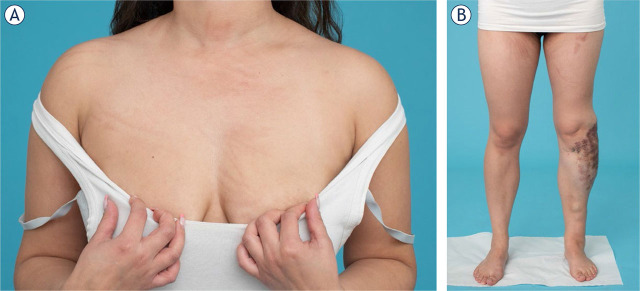
After Bleomycin ElectroScleroTherapy (BEST) flagellate dermatitis can occur on the sites of skin. **(A)** Scratching can cause permanent hyperpigmentation due to bleomycin. **(B)** Marks of the electrode insertion are visible at the site of the treatment.

- Most cases are performed under general anaesthesia due to the painful electric pulse sensation.
- Selected cases may be performed under sedation or local anaesthesia/block.
- Put ECG stickers on the sole of the foot and in axillae. ECG stickers have been associated with hyperpigmentation (Figure 1).
- Other skin fixations or their removal from the skin, such as eye taping, tube fixation or blood pressure cuff etc., may be associated with hyperpigmentation but are not contraindicated. Care should be taken to limit the amount of skin taping if possible. Endotracheal tubes can be tied, Blood Pressure (BP) cuffs should be placed over cotton wool. Removal should be undertaken with great care to avoid skin trauma (and thus hyperpigmentation). This can be facilitated by using a silicone-based spray to reduce the stickiness of the tape or plasters.
- ECG synchronisation system should be used when treating the left chest wall or close to the heart.
- After bleomycin administration keep FiO_2_ less than 30% if possible or as per local anaesthetic protocol. However, if there is any concern at any stage, oxygen should be administered as high as required but as low as needed.
- May consider ultrasound-guided analgesia injection of e.g. levobupivacaine or block, being aware of possible systemic drainage to reduce post-treatment pain.

### Route of bleomycin injection

- Direct percutaneous injection into the low-flow vascular malformation under ultrasound or other imaging guidance.
- In rare cases with multiple or extensive lesions, intravenous infusion can be considered. This requires a careful risk-benefit assessment in this scenario of treating a benign disease with a cancer drug.

### Preparations for the operative procedure

- Informed consent signed by the patient.
- Treatment area and laterality clearly marked (using US and marking the lesion on the skin).
- Pre-treatment urine pregnancy test in fertile female patients.
- WHO surgical safety checklist.
- Relevant imaging available in the theatre.

### BEST procedure

- Anaesthesia – as suggested.
- Bleomycin preparation and injection.
  - *Bleomycin has confusing unit nomenclature* and care should be taken to ensure predictable dosing. Historically, bleomycin dosage is described in terms of mg potency, where 1 mg-potency corresponds to 1 Unit or 1 000 International Units. Because 1 mg potency is not always equivalent to 1 mg weight, the International Unit measure is preferred.^24^
  - Take care: 1 mg potency translates nowadays to 0.56 – 0.66 mg weight of bleomycin sulphate, depending on local pharmacy preparation.
  - *The suggested concentration of bleomycin for injection is 1 000 IU/ml.* Bleomycin prepared as a solution at a concentration of 1 000 IU/ml dissolved in 0.9% NaCl. Bleomycin and other drugs should be clearly marked and distinguished on the operative tray.
    - For injection of the prepared bleomycin, dilute 1 part of this bleomycin solution with 3 parts of contrast medium (CM) providing a solution with a bleomycin concentration of 250 IU/ml for intralesional injection. When ultrasound guided injection only is performed, contrast is not needed. Bleomycin can be injected neat or foamed.
    - Bleomycin may be used foamed depending on local practice, e.g.: 1 ml albumin; 1 ml plain 1% lidocaine; 8 000 IU bleomycin; contrast agent may be added; orthogonal 3 way tap connection; 5 ml air- or according to local practice. It is also possible to dilute the mixture or bleomycin itself with normal saline to reduce the administered dose.
  - *Assessment of the extent of the vascular malformation.*
    - Prior to bleomycin injection assess the anatomy and extent of the vascular malformation either with ultrasound or fluoroscopy.
    - In bigger low-flow malformations (more than 5 cm in largest diameter) prior to bleomycin application, ultrasound-guided access to the vascular malformation should be obtained by positioning needles in all of the intended area: contrast agent injection through the needles and fluoroscopy or ultrasound should be performed to document the needle position, to assess the extent of punctured areas, to assess possible major venous drainage, and to assess the required volume of diluted bleomycin solution per areas. The aim is to fill the whole intended volume to be treated.
    - If necessary, drainage can be limited by compression, tourniquet or intravascular occlusion techniques (Lesion Puig type 3 to 4).
  - *Injected amount of bleomycin* depends on the size (longest diameter) of the malformation, drainage pattern, and dilution with contrast or other agents. Suggested doses for different longest diameters of malformations are:
    - Longest diameter below 1 cm - bleomycin dose up to 500 IU.
    - Longest diameter 1 – 3 cm – bleomycin dose between 500 – 1 000 IU.
    - Longest diameter 3 – 5 cm – bleomycin dose between 1 000 – 2 000 IU.
    - Longest diameter > 5 cm - bleomycin dose > 2 000 IU (maximum 5 000 – 10 000 IU).
    - *Maximal doses:* The cumulative dose in all treatments (lifetime dose) should normally not exceed 100 000 IU in an adult. In children, a cumulative total dose more than 1300 IU/kg, should not be exceeded.
    - *Maximal dose of bleomycin per session* delivered locally should normally not exceed 10 000 IU in adults, in children 200 IU/kg body weight
  - *Direct injection of bleomycin* into the malformation is verified by ultrasound, fluoroscopy or other imaging modalities. The bleomycin solution should be applied through the verified needle accesses in venous malformations and in macrocystic lymphatic malformations. Several needles or repeated injections could be used to ensure that the entire intended treatment area has been injected and filled. Interstitial or intravenous injection can be applied in microcystic lymphatic malformations.
  - *Intravenous administration* of bleomycin only for very large and/or multiple lesions or when there are too many compartments, or generally when local application is not possible. Intravenous bleomycin is infused over 5 minutes with a dose of 200 IU bleomycin per kg body weight, not exceeding 15 000 IU in total.
  - *Verification of the injection* either with ultrasound or fluoroscopy, possibly in 2 planes. If you see extravascular/interstitial contrast/bleomycin, you probably did not inject within the lesion and should repeat the treatment.
  - Electroporation of the low-flow malformation as recommended below.

### Electrode selection

- Electroporate the malformation, choosing the electrode according to the depth, extent and location of the malformation.
- Superficial cutaneous or subcutaneous malformations: Finger, linear or hexagonal electrodes.
- Deeper or larger surface area malformations: Single long needle electrodes (VGD) placed in a triangular geometry or other geometries with a maximum distance of 3 cm between them. The electric field should not exceed 1000 V per cm distance between the electrodes.

**TABLE 1.** Recommended preparation and dosing of intralesional injection (neat or foamed) of bleomycin for Bleomycin ElectroScleroTherapy (BEST)

	**Concentration**	**Preparation for injection**	**Maximal dose per session**	**Cumulative dose in all sessions**
**Bleomycin and contrast**	1 000 IU/ml in NaCl solution	1 part of solution in 3 parts of Contrast Medium (250 IU/ml)	10 000 IU in adults	100 000 IU in adults
			200 IU/kg in children	1 300 IU/kg in children-divided by number of sessions
			Divide the total dose into anticipated number of sessions	
			The interval between sessions should be at least 8 weeks	
**Foamed bleomycin**	1 000 IU/ml in NaCl solution	1 ml albumin; 1 ml plain 1% lidocaine; 8 000 IU bleomycin; contrast agent may be added; orthogonal 3 way tap connection; 5 ml air- or according to local practice	As above	As above

### Electroporation method

- Start the electroporation as soon as possible after intralesional drug administration. After systemic drug administration start electroporation within 8 minutes.
- Apply electric pulses to the area to be treated. If the area is larger than the area covered by the electrode, reposition the electrode. Multiple intralesional applications are needed for large lesions. As opposed to oncological therapy, very strict coverage by electroporation of the malformation or safety margins around the malformation are not required. It is strongly recommended to avoid overlapping of the treated areas, because of possible side effects due to potential irreversible electroporation, like swelling, bleeding, necrosis or hyperpigmentation.
- In venous malformations, electroporation should start from the point of drainage of the lesion towards the inflow of blood, to prevent outflow of bleomycin. Needle for direct bleomycin injection should be removed before applying electric pulses. Macrocystic lymphatic malformations are first drained, and then treated. Microcystic lymphatic malformations are treated covering the entire lesion if possible.

### Other considerations

- Careful consideration should be given to the possible risk of swelling after treatment, particularly in the head and neck area, airways, lips, eyelids, ears and genital areas. Significant swelling may occur with microcystic lymphatic malformations, particularly in the tongue.
- Patients must be advised to avoid skin trauma in the first 48 hours after the procedure (e.g. scratching, etc.) to avoid possible skin hyperpigmentation.

### Post-operative

- After the procedure, hospitalization according to the local procedure.
- Tongue or airway involvement: admit with High Dependency Unit / Intensive Therapy Unit (HDU/ITU) support as needed. This might include a prophylactic (pre-procedure) tracheostomy, prolonged intubation or in extreme cases an emergency tracheostomy.
- LMWH at local protocol discretion.
- Post-operative compression therapy at the discretion of the local protocol.
- Pain after treatment might be more intense when using a hexagonal vs linear or finger electrode due to more pulses being delivered.
- Local cooling may alleviate post-operative pain and swelling depending on the extent of the procedure.
- In case of acute pneumonitis due to bleomycin, administer high-dose corticosteroids (30 mg/kg) as soon as possible after bleomycin injection and onset of lung toxicity.
- Consider periinterventional antibiotic treatment after 20 or more applications of electric pulses (loss of skin barrier to bacteria due to puncture related skin trauma).
- Pain management according to local procedure (As an example for adult patients):
  - Basic analgesia with oxycodone 10 mg or oxycodone 20 mg twice per day with 12 h interval.
  - You can add etoricoxib 90 mg (or paracetamol/metamizole) once after 12 h.
  - In case of persisting moderate to severe pain (numeric rating scale [NRS], 0 = no pain, 10 = unbearable pain) NRS ≥ 4 in spite of oxycodone, add oxygesic 5 mg per os for max. 4 times/day every 2–3 h.
  - In case of further persistent pain (NRS ≥ 4), despite 4 times added oxygesic/day, increase oxycodone dose for 10 mg, but you should not exceed 2 × 40 mg oxycodone/day).
  - In case of pain < NRS 4, do not add oxygesic. Continue oxycodone until second post-operative day, thereafter, reduce and stop.

### Follow up

- 3–6 months unless a series of treatments are clearly going to be needed, then consider seeing earlier.

### Treatment interval

- At least 8–12 weeks in-between treatments.

## Discussion

The Current Operating Procedure (COP) for Bleomycin ElectroScleroTherapy (BEST), as outlined in this article, represents the first consensus-driven protocol for the treatment of low-flow vascular malformations. Multiple centers across Europe and the United Kingdom practice BEST, having acquired their skills either through treating tumours with electrochemotherapy—where standard operating procedures (SOPs) are already established—or by training under specialists in this field.^23^ This article summarizes the collective experience in a concise list of steps recommended to maximize safety and clinical effectiveness based on current knowledge (Figure 3, 4) The COP was initially developed by a small group of authors and subsequently reviewed and refined by a broader community of co-authors, ensuring comprehensive consensus within the BEST community. As most BEST applications have so far focused on treating low-flow vascular malformations, this COP is specifically tailored to this type of malformation. We anticipate that future iterations of the COP will evolve into SOPs based on more extensive evidence, ultimately facilitating the wider use and acceptance of BEST for both low-flow and high-flow vascular malformations.

**FIGURE 2.**
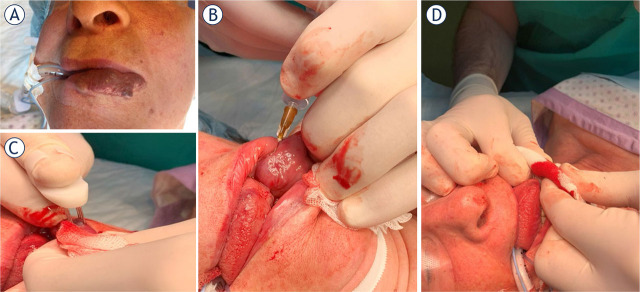
Procedure of Bleomycin ElectroScleroTherapy (BEST) in the treatment of low-flow vascular malformation on the lip. **(A)** Patient pre-treatment. **(B)** After the injection of bleomycin solution, the **(C)** electric pulses were applied on several areas with the finger electrode, avoiding overlap of the electric field. Procedure was completed in 10 minutes. The treated malformation was compressed **(D)** for several minutes to stop bleeding. Due to the vascular effects of BEST, the bleeding stopped spontaneously.

**FIGURE 3.**
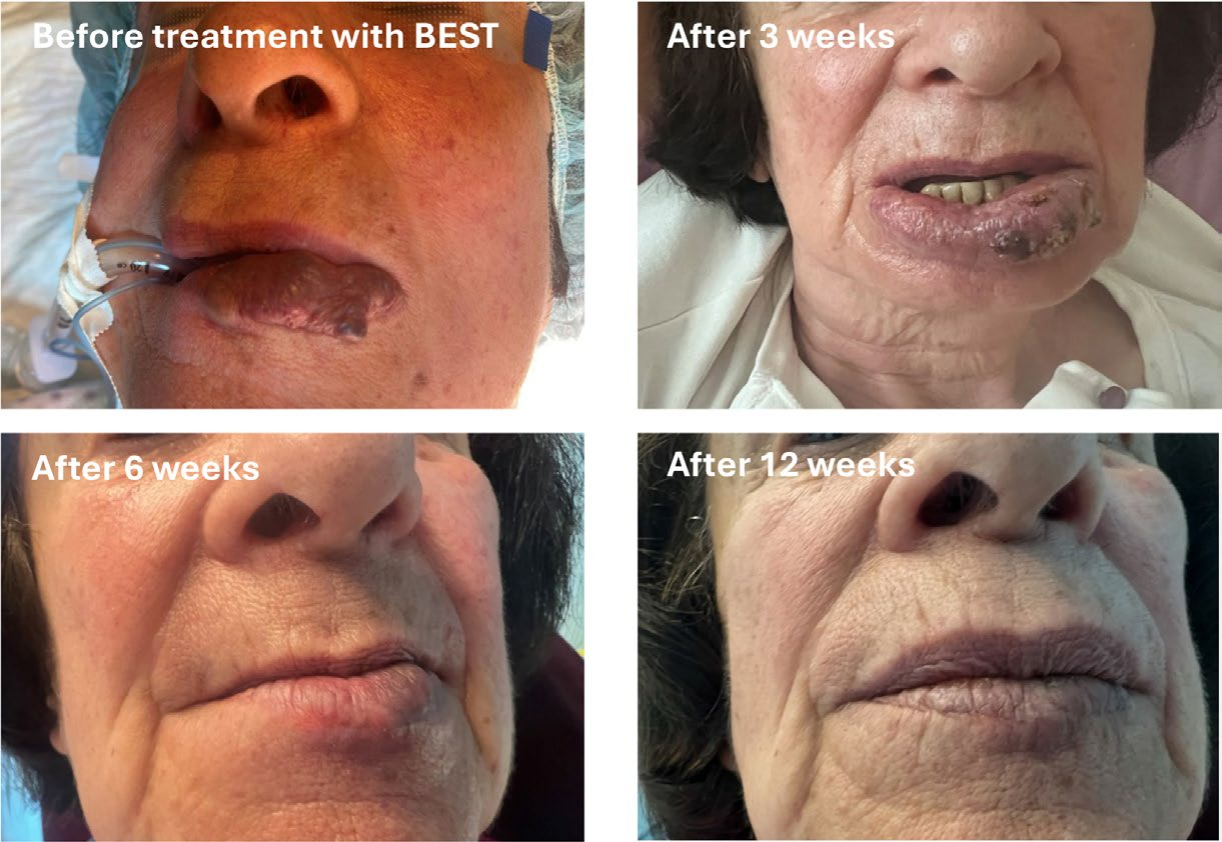
Example of Bleomycin ElectroScleroTherapy (BEST) treatment effectiveness on low-flow vascular malformation. After treatment, the treated area is oedematous, with a scab over the treated area after 3 weeks. The scab falls off in about 6 weeks with already visible treatment effectiveness. After 3 months, an excellent effect is visible after one treatment only.

**FIGURE 4.**
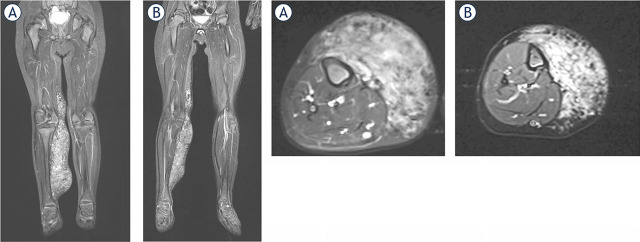
Microcystic lymphatic malformation before Bleomycin ElectroScleroTherapy (BEST) **(A)** and one year after **(B)**. A significant reduction of the malformation is observed.

The current understanding of BEST’s mechanism of action is derived from electrochemotherapy and its effects on the vascular system.^16^ Studies in mice, pigs, and humans have shown that tumour vessels are more sensitive to electrochemotherapy than normal vessels, likely due to the higher proliferation rate of endothelial cells.^17,25,26^ Bleomycin induces mitotic endothelial cell death, which may explain the differential effect observed between tumour and normal blood vessels.^27^ This concept can be extended to vascular anomalies, where various mutations in molecular signalling pathways lead to a higher proliferation rate of endothelial cells.^28,29,30,31,32^ Vascular malformations are influenced by several key molecular pathways. The PI3K/AKT/mTOR pathway is particularly significant, with mutations in PIK3CA commonly associated with venous and lymphatic malformations but can be also found in tumour endothelial cells. The RAS/MAPK pathway also plays a crucial role, with mutations in genes such as KRAS linked to various vascular anomalies.^33,34^ Additionally, the TIE2/TEK pathway, involving mutations in the TEK gene, is also known to contribute to the development of venous malformations. Both mutated signalling pathways are also found in tumours.^33,35,36^ Similarities in mutated genes between endothelial cells in tumour vessels and those in vascular malformations suggest a comparable phenotype and provide insights into the mechanisms of action of BEST in treating vascular malformations. Nevertheless, pathway activation in vascular malformations leads to localized, benign overgrowths of blood vessels, without the invasive properties of cancer. In cancer, activations of these pathways are part of a broader oncogenic process that not only promotes angiogenesis, but also supports tumour growth, metastasis, and resistance to therapy. Thus, to support the broader application of BEST and the development of evidence-based SOPs, more basic research is needed. Establishing preclinical models of vascular malformations in vitro and in animal models would offer deeper insights into the mechanisms of action of BEST and its clinical response.

Currently, the BEST procedure is based on the experience of a few centres, leaving several critical questions unanswered. These include, among other things, determining the optimal bleomycin dosage and the optimal interval between drug injection and electric pulse application. Current experience suggests that the required bleomycin dose for BEST is much lower than for electrochemotherapy, but further research is needed to establish the minimum effective dose.^1^ Additionally, the optimal number of applied electric pulses according to the area of vascular malformation and the coverage with the electric field to achieve a clinical response without over-treatment is still under investigation. Answering these questions requires preclinical models of vascular malformations and leveraging tools developed for electrochemotherapy, such as analytical methods for determining bleomycin concentration^37,38^, numerical models for simulating electric field distribution^39^, and modern radiological and molecular techniques for monitoring tissue and cellular changes.

When comparing BEST to other treatments for low-flow vascular malformations, several advantages and distinctive characteristics emerge. Surgical excision, while effective, often carries significant morbidity, including scarring and the risk of incomplete removal, leading to recurrence.^40^ Conventional sclerotherapy involves injecting sclerosing agents to induce fibrosis and shrinkage of the malformation, but it can be less effective in larger or more complex lesions and may require multiple sessions.^11,13,41^ Laser therapy is beneficial for superficial malformations but has limited efficacy in deeper or more extensive lesions and can cause skin discoloration or damage [42,43]. BEST, however, has so far demonstrated high effectiveness with fewer required sessions, leading to rapid and significant reduction in malformation size.^1^

As these questions are resolved, we will gain a clearer understanding of the fundamental principles of BEST. Subsequent steps will involve accumulating clinical data to assess the safety and effectiveness of BEST. This data will form the basis for developing SOPs, providing evidence-based guidelines enabling the safe and effective use of BEST to treat low-flow vascular malformations.
